# Severe Spasm of the Renal Artery after Blunt Abdominal Trauma Simulating End-Organ Infarction

**DOI:** 10.1155/2010/207152

**Published:** 2010-12-16

**Authors:** Adem Ucar, Aghakishi Yahyayev, Ayaz Agayev, Fatih Yanar, Selim Bakan, Mesut Bulakci, Tolga Akman, Ensar Yekeler

**Affiliations:** Istanbul University, 34452 Istanbul, Turkey

## Abstract

Traumatic occlusion of the renal artery is a serious injury. Management differs according to the grade of injury. In most circumstances, emergency surgical revascularization or endovascular intervention is required. We describe the case of a child with multiorgan injuries and spasm of the main renal artery after blunt trauma simulating arterial occlusion or end-organ infarction.

## 1. Introduction

Spasm of the renal artery is a complication that can be encountered during endovascular procedures [1]. Arterial spasm can also occur after blunt abdominal trauma and is considered to be secondary to contusion [2]. This condition should not be confused with traumatic occlusion of the renal artery due to thrombosis or intimal flap formation, which can cause devascularization of the kidney. Additional attention should be paid to distinguish between these two conditions because of the different therapeutic options available [3, 4]. In this contribution, we describe a case of severe spasm in the renal artery simulating end-organ infarction.

## 2. Case Report

**Figure 1:**
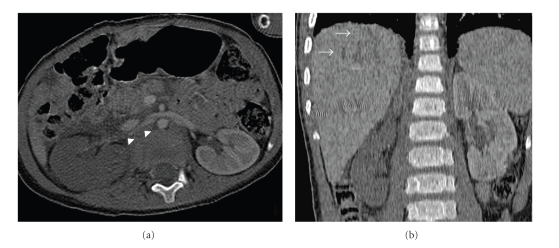
Axial curved reformatted image and coronal image of the abdomen showing normal proximal and middle segments of the renal artery (arrowheads) and no enhancement in the right kidney. Note the linear hypodensity in the liver compatible with laceration (arrows).

**Figure 2:**
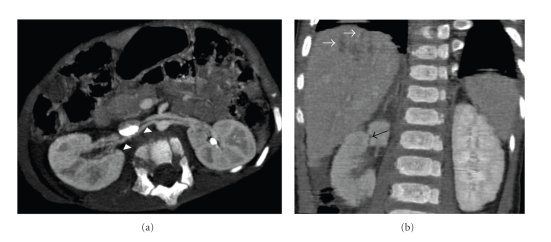
Axial curved reformatted image and coronal image of the abdomen demonstrating a patent renal artery with normal calibration (arrows) and opacification of the right kidney. Note laceration on the upper pole of the right kidney (black arrows) and in the liver (white arrows).

A 4-year-old child was brought to the emergency room after falling from the fourth floor of a building. CT of the chest and abdomen was undertaken within the first hours after the accident. Right pulmonary contusion with minimal effusion and multiple liver lacerations with mild perihepatic fluid were detected on CT. Enhancement was not observed in the right kidney. The proximal and middle portion of the right renal artery had narrowed lumens (Figures 1(a) and 1(b)). These findings were compatible with occlusion of the distal segment of the renal artery and end-organ infarction. The patient was hemodynamically stable, and laboratory findings were unremarkable, so endovascular management was planned. Control color Doppler ultrasonography 30 min before angiography detected normal vascularization of the right renal parenchyma and a patent renal artery. CT of the abdomen was obtained after 5 hours, and normal enhancement of the right kidney was confirmed (Figure 2). On 6-month followup, color Doppler ultrasonography was carried out, and vascularization of the kidney and renal artery was found to be within normal limits. 

Thus, complete recovery without complications was observed, and laboratory findings were normal. Nonopacification of the right kidney was considered to be due to spastic occlusion of the renal artery (presumably in response to trauma).

## 3. Discussion

Renal injuries are classified into five grades according to the increasing severity of trauma. This classification system recognizes the progressive nature of parenchymal and vascular damage according to the increasing level of trauma [5].

Devascularization of the entire kidney due to vascular laceration or thrombosis of the renal artery is considered to be the most severe form of renal injury (grade 5). Such injuries may occur with or without parenchymal lacerations. If the kidney is devascularized as a consequence of an isolated intimal injury to the renal artery that results in thrombosis, extensive retroperitoneal hemorrhage and hematuria may be absent [6].

The management of renal trauma ranges from observation without intervention in minor lacerations to an emergency laparatomy for hemodynamic compromise. Minimally invasive techniques are being adopted to manage significant renal trauma if hemodynamic compromise is absent. As with all solid-organ injuries in children, there has been a shift to conservative management (if possible) [3, 4]. 

The classic findings of traumatic renal infarction on CT include nonenhancement of the kidney on the affected side, retrograde opacification of the renal vein from the inferior vena cava, and abrupt truncation of the renal arterial lumen at the point of occlusion. The cortical rim nephrogram sign of a devascularised kidney may be absent in the acute setting [6]. Nonopacification of one or both kidneys on CT may be due to spasm of the renal artery secondary to contusion. [2]. This condition should not be confused with traumatic occlusion of the renal artery. In our case, the right kidney of the patient did not show enhancement. Taking into account that severe renal artery spasm and traumatic occlusion of the renal artery may occur simultaneously, making the correct interpretation was difficult. 

Renal artery spasm is a known complication of renal angioplasty during endovascular procedures. Ogita et al. reported multiple spasms of renal arteries after percutaneous transluminal renal angioplasty in children [7]. 

Spasm of the renal artery can also be seen after abdominal surgery in response to external stimuli. Yamagiwa et al. reported atrophy of the kidney following removal of the neuroblastoma. This was presumably due to renal artery spasm and endothelial damage, which probably led to stagnation of renal blood flow and finally to thrombosis of the renal artery [8]. Koehler and Friedenberg demonstrated three cases of renal artery spasm during angiography simulating end-organ infraction [9].

## 4. Conclusion

Traumatic injuries to the renal artery and severe spasm of the renal artery secondary to contusion may produce identical findings on CT. Correlation between clinical findings and laboratory results has an important role in such situations. Repeated radiological assessment is therefore recommended to avoid misinterpretation. To reduce the risks of inappropriate treatment, radiologists must be aware of the CT findings of blunt trauma to the kidney.
